# Ovarian plasmacytoma: a case report

**DOI:** 10.11604/pamj.2023.44.108.37603

**Published:** 2023-02-27

**Authors:** Anass Haloui, Nassira Karich, Nada Akouh, Noura Seghrouchni, Younesse Najioui, Asmae Aissaoui, El Mehdi Tiabi, Samia Malki, Samah Tahri, Houda Bachir, Abbou Widad, Rim Ouajdi, Imane Kamaoui, Mhand Mohammed, Badr Serji, Tijani Elharroudi, Amal Bennani

**Affiliations:** 1 Laboratory of Pathological Anatomy, Mohammed VI University Hospital, Faculty of Medicine and Pharmacy of Oujda, Mohammed First University, Oujda, Morocco,; 2 Department of Internal Medicine, Mohammed VI University Hospital, Faculty of Medicine and Pharmacy of Oujda, Mohammed First University, Oujda, Morocco,; 3 Department of Radiology, Mohammed VI University Hospital, Faculty of Medicine and Pharmacy of Oujda, Mohammed First University, Oujda, Morocco,; 4 Department of Surgical Oncology, Regional Oncology Center, Mohammed VI University Hospital, Faculty of Medicine and Pharmacy of Oujda, Mohammed First University, Oujda, Morocco

**Keywords:** Extramedullary plasmacytoma, ovary, plasma cell neoplasm, case report

## Abstract

Plasmacytomas are a rare spectrum of plasma cell neoplasms that are single localized tumours, lacking the clinical features of plasma cell myeloma with no radiographical evidence of additional plasma cell tumours. Two clinical variants of plasmacytomas can be distinguished: solitary plasmacytoma of bone and extramedullary (or extraosseous) plasmacytoma. The latter is rare, representing 1% of all plasma cell neoplasms, occurring most frequently in the upper airways. Ovarian localization is exceptional, with only a few cases being reported in the literature. We herein report a case of an ovarian extramedullary plasmacytoma occurring in a 56-year-old woman who consulted for abdominal pain and abdominal mass, while highlighting the main histological and immunohistochemical features of this rare malignancy, along with a thorough review of literature gathering all cases of ovarian plasmacytomas reported to date.

## Introduction

Extramedullary plasmacytomas are a group of neoplasms resulting from the clonal expansion of mature plasma cells, located in tissues other than bone. They represent 1% of plasma cell neoplasms and occur most often in the upper airways. Ovarian plasmacytomas are an exceptional presentation, with only rare cases reported in the literature. We herein present a case of ovarian plasmacytoma, while highlighting the main histological and immunohistochemical features along with a thorough review of literature, regarding presentation and diagnostic workup.

## Patient and observation

**Patient information:** a 56-year-old woman with a history of breast neoplasia for which she had a mastectomy, consulted for abdominal pain.

**Clinical findings:** physical examination revealed abdominal tenderness and an abdominal mass.

**Timeline of the current episode:** on October 2021, an abdominal-pelvic computed tomography (CT) scan was performed revealing a right ovarian mass, along with a normal biological workup. A few days after, the patient underwent a right adnexectomy.

**Figure 1:**
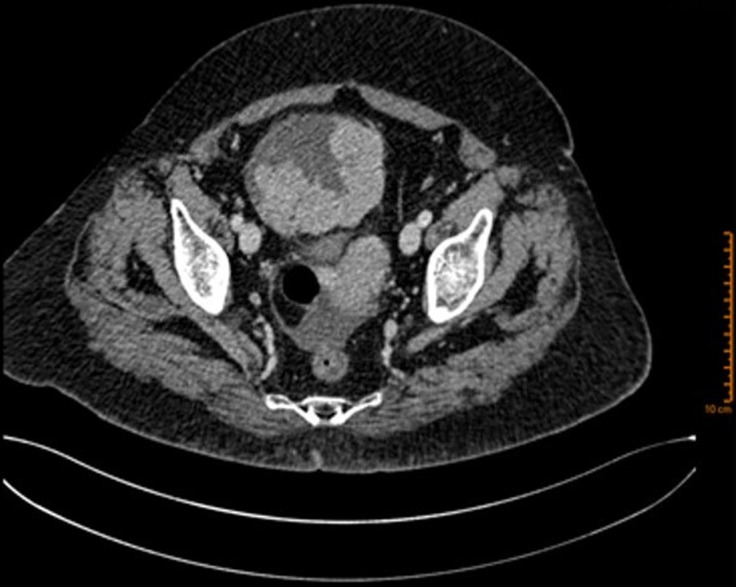
abdominal-pelvic CT scan showing an oval, well-limited right ovarian mass, with a prevailing cystic component and a solid component enhanced after injection of contrast agent

**Figure 2:**
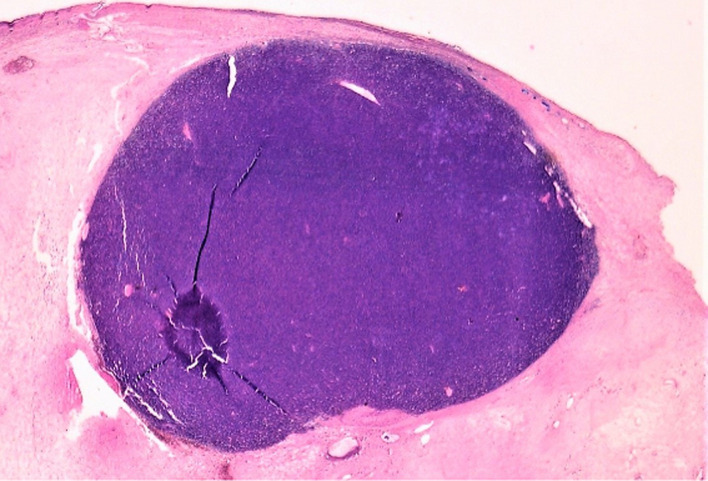
low power view showing a tumoral proliferation arranged in a well limited nodule within the ovarian cyst´s wall (hematoxylin and eosin stain, magnification x4)

**Figure 3:**
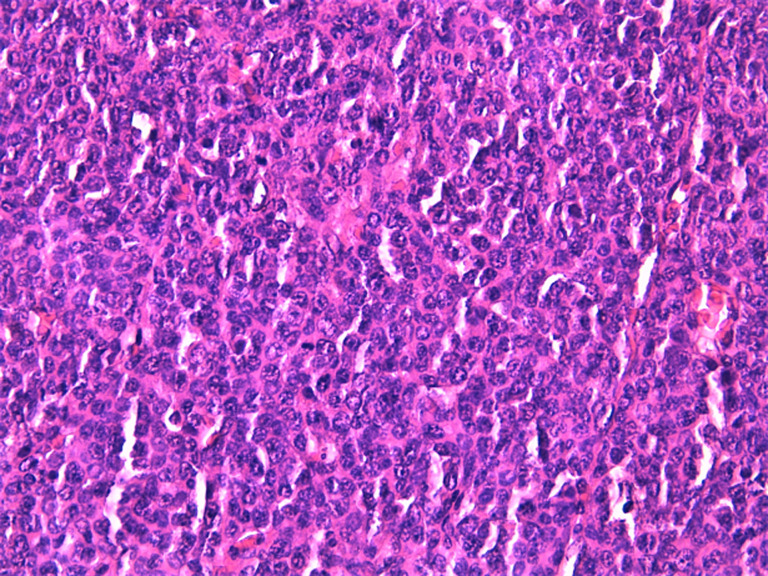
high power view showing diffuse sheets of tumoral cell displaying a plasmacytoid morphology, with rounded eccentric nucleus, coarse clumped chromatin, and abundant eosinophilic cytoplasm (hematoxylin and eosin stain, magnification x40)

**Figure 4:**
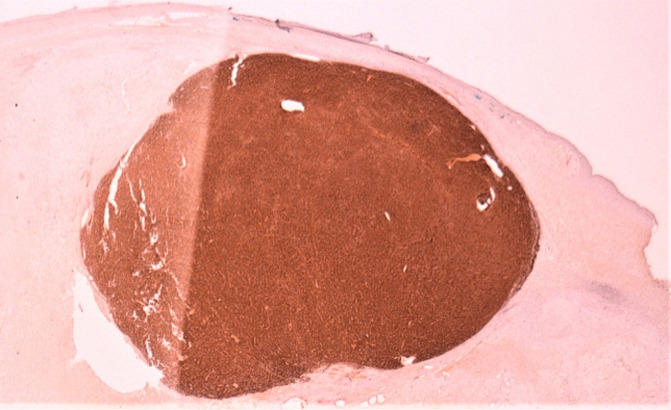
low power view demonstrating diffuse expression of CD138 by the tumoral cells (magnification x4)

**Figure 5:**
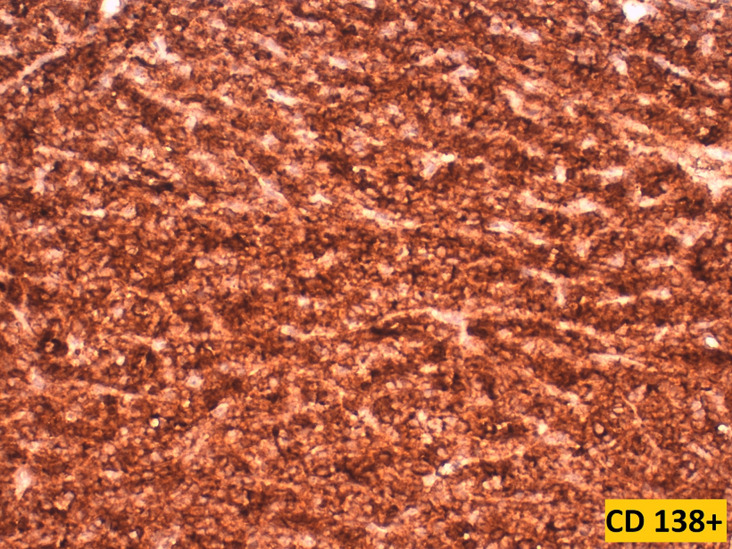
high power view showing strong and diffuse membranous expression of CD138 by tumoral cells (magnification x40)

**Figure 6:**
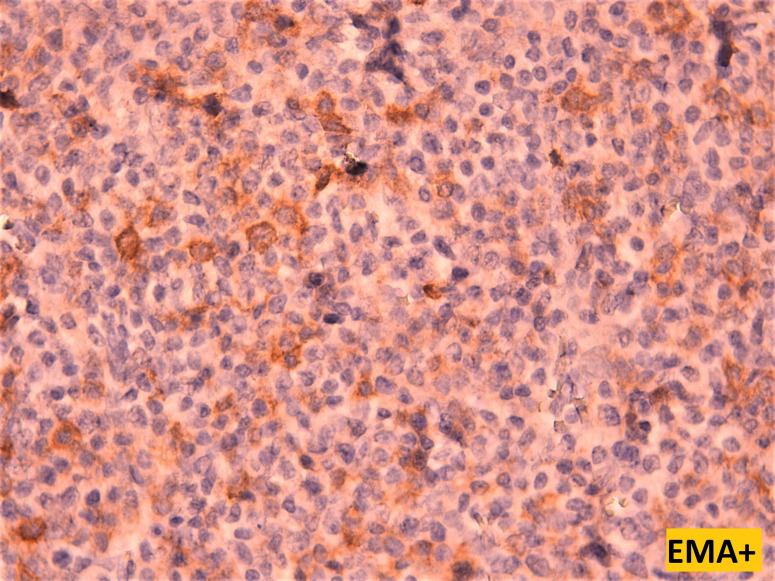
high power view showing patchy membranous and cytoplasmic staining by EMA of the tumoral cells (magnification x40)

**Figure 7:**
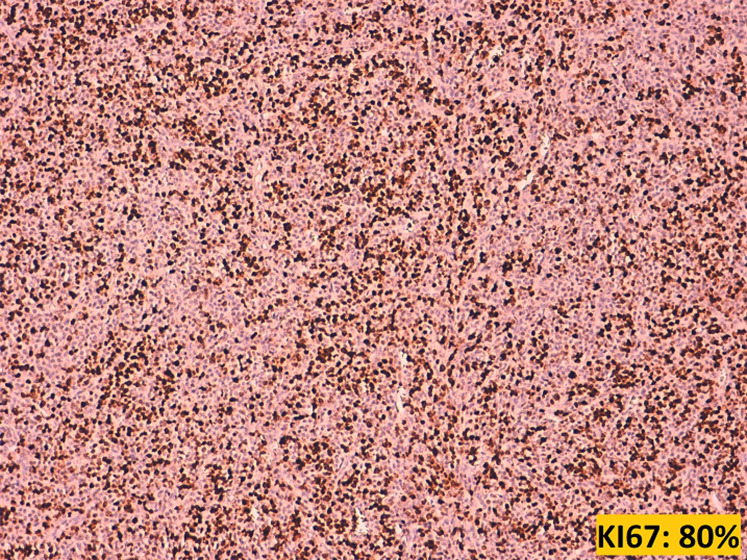
Ki67 estimated at 80%

**Diagnostic assessment:** the abdominal-pelvic CT scan revealed a right ovarian mass, displaying a prevailing cystic component and a solid component enhanced after injection of contrast agent (Figure 1). The biological workup was normal, including the absence of a monoclonal gamma peak on protein electrophoresis, and a bone marrow plasmacytosis estimated at 2%. A right adnexectomy was performed. Macroscopically, the ovarian mass measured 20 x 12 x 7 cm, showing a solid and multilocular cystic appearance at the cut, with yellowish serous content. The wall of the cysts was the site of multiple scattered whitish nodules. Histological examination showed a tumoral proliferation arranged in well-limited nodules (Figure 2), made of sheets of tumoral cells showing a plasmocytic morphology, with abundant eosinophilic cytoplasm and rounded eccentric nucleus with coarsely clumped chromatin (Figure 3). The tumoral cells showed diffuse membranous expression of CD138 (Figure 4, Figure 5), as well as an expression of epithelial membrane antigen (EMA) in a more heterogeneous manner (Figure 6). Ki67 was estimated at 80% (Figure 7).

**Diagnosis:** the diagnosis of ovarian extramedullary plasmacytoma was sustained, based on morphological and immunohistochemical features, after ruling out a plasma cell myeloma.

**Therapeutic interventions:** the patient underwent a right adnexectomy.

**Follow-up and outcome of interventions:** to date, the patient remains asymptomatic and in excellent clinical condition, with no reported complications.

**Patient perspective:** the patient remarked “I feel well”.

**Informed consent:** the patient gave informed consent.

## Discussion

Plasmacytomas are a group of neoplasms resulting from a clonal expansion of mature plasma cells, typically synthesizing monoclonal immunoglobulin and causing a monoclonal gammapathy. They usually lack the clinical features of plasma cells myeloma, with no clinical or radiological evidence of additional plasma cell tumors. Two clinical variants of plasmacytomas can be distinguished: solitary plasmacytoma of bone and extramedullary (or extraosseous) plasmacytoma [1]. Extramedullary plasmacytoma is rare, accounting for 1% of all plasma cell neoplasm. They occur most commonly in the upper aerodigestive tract, but other localizations are possible such as lymph nodes, bladder, breasts, thyroid, testes, parotid glands, skin, and central nervous system [2]. The ovarian localization remains exceptional. Only a few cases of ovarian plasmacytomas have been reported in literature, all of them being reported as single cases. In our review of the bibliography, we have listed 18 cases, including our present case (Table 1) [2-20].

**Table 1:** cases of ovarian plasmacytomas reported by literature in chronological order

Author	Age	Symptoms	Ovary	Size	Macroscopic appearance	Immunohistochemistry
Voght, 1938	30		Unilateral	Size of a fist		
Bambirra, 1982	44	Abdominal pain	Bilateral	R 14.3 x 5.3 x 4 cm ; L 12.3 x 9 x 36 cm		
Hautzer, 1984	56	Abdominal mass	Left	24 x 14 x 11 cm		
Talerman, 1987	35	Abdominal mass	Unilateral	15 x 12 x 9 cm		
Cook, 1988	63	Abdominal pain, distension abdominal mass	Left	12 x 10 x 7 cm	Smooth outer surface; cut surface solid and lobulated, pale yellow	Alpha heavy chains+, kappa light chains+
Andze, 1993	12	Pelvic mass	Left	12.3 x 8 x 3.8 cm		
Emery, 1999	54	Abdominal swelling and tenderness	Left	15 x 12.5 x 7.6 cm	Smooth outer surface; on cut section, the ovarian parenchyma is replaced by a soft, tan, neoplasm with focal areas of hemorrhage	CD43+, kappa light chains+, CD20-, CD45- , lambda immunoglobulin light chains -
Ben Salah, 2011	36	Pelvic mass	Bilateral	20x 18 x 16 cm		
Naourez, 2011	36	Abdominal pain, abdominal, distension and metrorrhagia	Bilateral	Left: 35 cm; right: 30 cm	Solid at cut, with lobulated appearance; tumor capsule was intact	CD138+, lambda light chains+
Zhong YP, 2012	54	Abdominal pain	Right	12 x 12 x 10 cm	The tumor capsule was intact, with thick walls; gray color	CD38+, CD138+, CD45+, CD99+, Ki-67: 90%
Shakuntala, 2012	35	Abdominal mass and intermittent pain	Right	14 x 13.5 x 6 cm	Solid and cystic areas	CD138+, CK+, Lambda light chains+, EMA focally +; negative for CD79a and kappa light chains
Feldman, 2015	84	Weight loss, constipation, dizziness, palpitation, loss of appetite; abdominal mass	Right	14 cm	Smooth capsule focally calcified; cut surface shows multiple cysts with myxoid, rubbery and hemorrhagic septa and contents	CD138+, kappa light chains+; CK-, CD20-, CD3-, CD5-, BCL1-PAX5-
Mondal, 2015	47	Abdominal pain	Left	10 x 9.5 x 6 cm	Exclusively cystic with yellowish content; thickness of cyst wall is 2.1 cm; inner wall showed multiple tiny nodules and striations; necrotic material was seen; within the thickened portion of cyst wall	CD138+, CD38+, CD45+, CK-, inhibin-, synaptophysin, desmin-, CD99-
Tomaselli, 2016	46	Abdominal mass and pain	Right	15x14x10cm	Very vascular ovarian mass with solid cystic areas	CD138+, lambda light chains+, CD20-
Cardenas, 2020	48	Abdominal pain	Bilateral	10 cm each		
Fares, 2021	52	Ovarian mass detected at pelvic ultrasound for recurring episodes of metrorrhagia	Left	4 x 3.5 x 3 cm	Well-circumscribed, encapsulated, and white tumor	CD138+, EMA+, lambda light chain restriction; CK-, SMA-, inhibin- H-caldesmone-; Ki67: 10%
El amouri, 2022	42	Abdominal distension	Left	18 x 8 x 5 cm	Grayish-brown solid surface with some cystic changes; smooth outer surface	CD138+, CD45+, CD79a focal+; kappa light chains+; CD20-, CD3-, MPO-, Inhibin-CK-, CD68- Ki67: 40%
Present case, 2022	56	Abdominal mass and pain	Right	20 x 12 x 7 cm	Solid and multilocular cystic appearance at cut; yellow whitish, yellowish serous content	CD138+; EMA+ heterogenous; CK-, Chromogranin- inhibin- CD20-, PAX5-, CD3-

The age of presentation is quite variable, ranging from 30 to 84 years old, with a mean age of 46 years. Only one case affecting a 12-year-old child was described [3]. The left ovary appears to be slightly more affected, although cases with bilateral localization have also been reported. When comparing all cases reported in the literature including our case, 7 cases involved the left ovary, 5 cases involved the right ovary, and 4 other cases were bilateral. Only two cases were reported as unilateral, with no data regarding the laterality of the tumor [4,5]. Clinically, patients usually present with symptoms related to ovarian mass, such as pelvic pain and abdominal mass. The tumor is usually diagnosed at a large size. The largest reported size was a 35 cm long axis, while the minimal reported size was a 4 cm long axis, which is somewhat reminiscent of the first case reported by Voegt in 1938 who described the tumor as the size of a fist [5]. The large size at the time of the presentation is more likely due to the insidious progression of this tumor. In our case, the patient complained of abdominal pain with an abdominal mass on physical examination. The abdominal CT scan revealed a right ovarian mass measuring 20 x 12 x 7 cm, which is consistent with the literature data.

Ovarian plasmacytoma generally presents as a mass with a smooth outer surface, usually with a thick capsule, which may focally be calcified. At incision, the ovarian parenchyma can be partially or completely replaced by a lobulated solid mass of soft consistency, ranging in colors from tan to yellow, to white or gray, brown. It can also be exclusively cystic with thick walls, or have a dual component, with both solid and cystic areas. Hemorrhagic, necrotic, and myxoid areas may also occur (Table 1). In our case, the mass displayed a solid and multiloculated cystic appearance on a cut, of yellowish-white color, with a serious yellowish content.

The histological appearance of the tumoral proliferation was broadly consistent in all cases reported in literature, with tumor cells displaying a plasmacytoid morphology with an eccentric nucleus and abundant cytoplasm. The main issue remains to think of a plasmacytoma at first glance. Since ovarian localization of this tumor is relatively rare, pathologists may be reluctant to make this diagnosis based on morphology alone and would be more tempted to rule out the most common diagnoses first, such as a granulosa tumor. Therefore, the definitive diagnosis of ovarian plasmacytoma has always been made after immunochemistry, along with a correlation of clinical, radiological, and biological findings.

Plasma cell differentiation can be evaluated by the expression of CD138, CD38, CD79a and MUM1. A further characteristic is the strong expression of restricted cytoplasmic light chains (kappa or lambda) in almost all cases, which can be useful in distinguishing a monotypic plasmacytoma from polytypic reactive plasma cell infiltrates. Usually, mature neoplastic plasma cells will not express pan-B-cell markers, such as CD20, PAX5. However, CD20 can be expressed in 20% of plasma cell myeloma, and Cyclin D1 is positive in cases that harbors t (11;14) and in some case with hyperdiploidy [6]. Unlike normal plasma cells, CD45 is usually negative or expressed at low levels, while CD56 can be seen in 70% of plasma cell myeloma [7]. The immunophenotype of extramedullary plasmacytomas appears to be similar to that of plasma cell myeloma. However, extraosseous plasmacytoma typically lacks the expression of CD56 and cyclin D1. In our literature review, we noticed that several different panels were used, probably depending on the initial suspicion of the pathologist when confronted with a tumor proliferation made of cells of such a monomorphic appearance. CD138 was widely used to confirm plasmacytoid differentiation of the tumor cells, along with CD38 in two cases, and CD79a in one case. EMA was reported to be focally expressed in 3 cases, including ours. CD45 when performed, was positive in 3 cases, and negative in two cases. EMA was reported to be focally expressed in 3 cases, including our own case. Kappa light chains and lambda light chains were respectively positive in 4 cases each (Table 1).

Since extramedullary plasmacytomas share with multiple myeloma the same morphological appearance of the tumor cells, and their immunohistochemical profile is relatively similar, the distinction requires a variety of additional clinical, biological, and radiological arguments. Diagnostic criteria for extramedullary plasmacytoma include histologic confirmation of plasma cell proliferation, bone marrow plasmacytosis of less than 5% on bone marrow biopsy specimens, the absence of clinical features of multiple myeloma, and normal bone imaging [6]. The distinction with multiple myeloma is important since more than 60% of patients who are treated for solitary plasmacytoma are cured with only local therapies, while the 5-year survival for patients with plasma cell myeloma is around 35% [8]. Progression to multiple myeloma is the most feared complication, occurring in about 15% of cases, particularly in the setting of minimal bone marrow involvement [6]. Regional recurrences develop in as many as 25% of cases, with the possibility of distant extraosseous metastasis. Thus, active surveillance afterward is indicated. The National Comprehensive Cancer Network (NCCN) guidelines recommend regular complete blood pictures, serum chemistries (LDH, albumin, calcium, beta 2 microglobulin), serum immunoglobulins, serum and urine-free light chain assays, and imaging [9].

## Conclusion

Extramedullary plasmacytomas in the ovary remain rare but possible neoplasms, justifying a meticulous morphological examination in the quest for plasmacytoid differentiation, particularly when confronted with a monomorphic tumoral proliferation with eccentric nuclei. The incorporation of plasma cell markers along with the immunoglobulin light chain in the immunohistochemistry panel for such ovarian proliferations can be of great help in challenging cases. It is of utmost importance to eliminate bone marrow plasmacytosis and bone involvement that may be part of multiple myeloma, given the therapeutic and prognostic disparity implied.
